# Evaluation of predictors indicating paroxysmal atrial fibrillation in patients with acute ischemic strokes: the Find-AF_RANDOMISED_ trial

**DOI:** 10.1186/s42466-026-00471-x

**Published:** 2026-02-23

**Authors:** Mark Weber-Krüger, Antonia Zapf, Evgeny Protsenko, Jan Liman, Gerhard F. Hamann, Pawel Kermer, Katrin Wasser, Timo Uphaus, Sonja Gröschel, Klaus Gröschel, Rolf Wachter

**Affiliations:** 1 Clinic for Palliative Care, University Medicine Göttingen, Göttingen, Germany; 2 Institute for Medical Biometry and Epidemiology, University Medical Center Hamburg-Eppendorf, Hamburg, Germany; 3 Clinic for Neurology, Nordwestkrankenhaus Sanderbusch, Sande, Germany; 4 Clinic for Neurology, Klinikum Nürnberg, Nürnberg, Germany; 5 Gerhard F. Hamann, Independent, Wiesbaden, Germany; 6 Clinic for Neurology, University Medicine Göttingen, Göttingen, Germany; 7 Clinic and Policlinic for Neurology, University Medicine Mainz, Mainz, Germany; 8 Clinic and Policlinic for Cardiology, University Medicine Leipzig, Leipzig, Germany

## Abstract

**Background:**

Detecting concealed paroxysmal atrial fibrillation after stroke requires elaborate electrocardiographic monitoring. We evaluated previously established predictors to quantify the individual risk of detecting atrial fibrillation within six months in the Find-AF_RANDOMISED_-trial.

**Methods:**

We analyzed 200 patients ≥ 60 years with acute ischemic strokes in the intervention arm of the Find-AF_RANDOMISED_-trial. Patients received three ten-day Holter-electrocardiograms within six months. Regression analyses and receiver-operator-characteristics were used to select promising biomarkers and assess predictive performance. We applied previously established cut-offs for the most promising markers to determine those at a high risk of underlying atrial fibrillation.

**Results:**

27/200 patients (13.5%) had atrial fibrillation after six months. The left atrial diameter, atrial premature beats, supraventricular runs and brain natriuretic peptide were associated with atrial fibrillation, whereas the established markers age and suspected stroke etiology were not. Atrial premature beats differentiated best between those with and without atrial fibrillation (area-under-the-curve = 0.75). Only brain natriuretic peptide ≥ 100pg/ml and supraventricular runs ≥ 20 beats independently predicted atrial fibrillation in multivariable models.

**Conclusions:**

Supraventricular runs and brain natriuretic peptide were the most promising predictors to define a high risk of underlying atrial fibrillation after stroke in our study. Future screening strategies for atrial fibrillation in stroke patients should focus on these parameters rather than the suspected stroke etiology.

**Trial registration:**

clinicaltrials.gov NCT01855035 registered 05132013 https//www.clinicaltrials.gov/study/NCT01855035?tab=table.

**Supplementary Information:**

The online version contains supplementary material available at 10.1186/s42466-026-00471-x.

## Background

Atrial fibrillation (AF) is a common cause of cerebral ischemia [45] and the diagnosis of electrocardiogram (ECG)-detected AF usually changes secondary preventive medication from platelet inhibitors to oral anticoagulation [18]. In addition to oral anticoagulation, stroke patients with manifest AF also benefit from early heart rhythm control [17]. However, this approach is not routinely considered by clinicians [15].

While sustained AF is easily identifiable by 12-lead-ECG or telemetry, paroxysmal AF (pAF) often remains concealed, and its detection requires elaborate prolonged ECG-monitoring. However, all types of enhanced ECG-monitoring (Holter-ECGs, external or implanted event-recorders) have specific disadvantages: some are cumbersome to wear, others require elaborate analyzation, most are associated with relevant costs; implanted devices require a small surgical procedure, limiting widespread application in clinical practice. The importance of identifying underlying pAF is emphasized by the fact that preventive benefits of oral anticoagulation in patients with manifest AF [31, 35] are outweighed by bleeding complications of empiric anticoagulation in stroke patients without AF. This also applies to patients with “atrial cardiomyopathy” [16], i.e. showing signs of structural or electric atrial alterations and patients with “embolic strokes of unknown source” (ESUS), i.e. patients with ischemic stroke patterns indicative of an embolic cause [2].

Considering the limited capacities of complex prolonged ECG-monitoring on the one hand and the benefits of oral anticoagulation being restricted to those with manifest AF on the other hand underlines the importance of defining valid AF-predictors to focus elaborate ECG-procedures on those with a high risk of concealed AF.

A consensus document by the German Heart and Brain working group [11] listed several predictors aimed to classify the risk of underlying AF in stroke patients. These include advanced age, echocardiographic markers of left atrial enlargement (left atrial diameter, LAD), increased natriuretic peptides and enhanced supraventricular ectopic activity (ESVEA, i.e. frequent atrial premature beats (APB) or prolonged supraventricular (SV-)runs [6]. The authors also attributed a high risk of underlying AF to those with stroke etiologies (based on the “TOAST”-classification [1] associated with territorial stroke patterns on cerebral imaging, i.e. non-AF-related cardioembolic strokes and those related to large artery atherosclerosis as well as cryptogenic strokes, including those fulfilling the ESUS classification. Meanwhile they acknowledge uncertainties regarding the implications of brain lesion patterns and suspected etiology with the likelihood of underlying AF.

So far, comparative analyses of these AF predictors have not been performed in larger studies, mostly because few studies provide data for all parameters.

Find-AF_RANDOMISED_ is a randomized multicenter trial that investigated the yield of newly diagnosed AF in stroke survivors aged ≥ 60 years. In the intervention arm, patients received ten-day Holter-ECG monitoring (shortly after randomization and after three and six months) versus usual care, i.e. ECG-monitoring according to local standards. The primary endpoint was newly detected AF after six months.

In the current analysis, we aimed to compare the predictive value of different AF-predictors to guide stroke physicians which parameter to use to distribute limited enhanced ECG-monitoring capacities.

## Methods

*Study design*: Find-AF_RANDOMISED_ (NCT01855035) was a randomized and controlled multicenter trial. The trial design [44], primary endpoint [38] and data from the three-year follow-up [39] have been published. In brief, patients ≥ 60 years with acute ischemic strokes but without known AF were included. They were randomized 1:1 to either extended Holter-ECG-monitoring or standard-of-care. Patients received cerebral imaging, vascular imaging and echocardiography as indicated by the treating physician. All data were collected prospectively, following predefined case report forms. Blood samples were collected for biomarker analyses of which, meantime, brain natriuretic peptide (BNP) has been reported as a valid AF predictor [41]. The trial complies with the Declaration-of-Helsinki and was approved by all local ethic committees. All patients (or their legal representatives) provided written and informed consent.

*ECG-monitoring and analysis*: AF was defined according to AF-guidelines [8], including only typical episodes lasting ≥ 30 s. Patients in the intervention-arm received three times ten days of Holter-ECG-monitoring, using a commercially available external five-lead Holter-ECG-recorder (CardioMem 3000, getemed, Teltow, Germany). The study Holter-ECGs were analyzed by a core-laboratory using the applicable software (CardioDay, getemed, Teltow, Germany) and following a predefined standard-operating-procedure. The number of APB and the longest SV-run were determined within the first 24-hour interval, unless it contained an episode of AF or was of insufficient quality, in which case the first evaluable day was chosen. APB were detected by the software’s automatic analysis algorithm and were manually revised. SV-runs were counted manually.

*Brain imaging*: All patients received cerebral imaging, i.e. computer tomography (CT) or magnetic resonance imaging (MRI) as indicated by the treating physician. ESUS criteria were not evaluated in this trial.

*Cardiac imaging*: Patients received echocardiography during clinical routine, which was not a mandatory part of the trial. However, investigators were encouraged to collect as many data as possible, including LAD, but also left atrial volume index (LAVI) and tissue Doppler measurements indicating atrial dysfunction (a’), summarized in the index LAVI/a’, which had shown incremental diagnostic value in previous analyses [27, 30] .

*Natriuretic peptides*: Blood samples were collected at baseline and at the three-month-visit. They were initially stored at ≤ -20 °C at the local site and were then transported to the coordinating study site in Göttingen, where they were stored at -80 °C, before being analyzed. The BNP levels were determined using a sandwich chemiluminescence immunoassay on the ADVIA Centaur (Bayer Diagnostics, Munich, Germany).

*Definition of the suspected stroke etiology*: The most likely etiology of the occurred stroke was determined by the treating neurologist by means of the TOAST classification.

*Predefined cut-offs*: We evaluated cut-offs proposed in a published consensus document [11]. It recommends to use age (low risk: <60 years, high risk: ≥75 years), APB (low risk: <120/day; high risk: ≥480/day), SV-runs (low risk: <5 beats, high risk: ≥20 beats), BNP (low risk: <50pg/ml; high risk: ≥100pg/ml), LA-diameter (low risk: <40 mm; high risk: ≥45 mm) and suggested etiology (low risk: small artery occlusion (SAO), other etiology; high risk: large artery atherosclerosis (LAA), cryptogenic stroke (including ESUS), other cardioembolic (CE) etiology), to define the underlying risk of AF.

*Statistics*: Continuous baseline variables were compared between patients with and without AF during follow-up by median, quartiles, and Mann-Whitney-U-test. Categorical data were compared by absolute and relative frequencies, and Chi²-test or Fisher’s Exact test. For all parameters that showed a relevant difference here, we performed receiver-operator-characteristics (ROC)-analyses and calculated the area-under-the-curve (AUC). Using the pre-defined cut-off levels, we calculated the sensitivity, specificity, positive-predictive-value and negative-predictive-value and performed a multiple logistic regression model (in a first step a full model, in a second with stepwise selection using categorical variables clustered by the predefined cut-offs). Statistical analyses were performed using IBM SPSS Statistics 29.0 (IBM, Armonk, USA), the logistic regression models were analyzed using SAS Version 9.4.

## Results

A total of 402 stroke patients were included in the Find-AF_RANDOMISED_ trial. Of those, 200 were randomized to the intervention arm and yield the study population of this analysis. Of those, 27 (13.5%) were diagnosed with novel AF after six months.

 Table 1 shows the baseline characteristics of those with versus without newly detected AF and the availability of data for each AF-predictor. 69 (34.5%) were < 70 years and 31 (15.5%) were ≥80 years old.

**Table 1:** Baseline characteristics

Age, years, median (inter-quartile-range, IQR)	no AF (number, *n* = 173)	AF (*n* = 27)afAF (*n* = 27)	*p*-value
	72 (65;76)	73 (65;80)	0.453
Female gender, n (%)	70 (40.5%)	15 (55.6%)	0.140
History of			
Stroke, n (%)	28 (16.2%)	6 (22.2%)	0.437
Transient ischemic attack, n (%)	12 (6.9%)	1 (3.7%)	0.526
Heart failure, n (%)	8 (4.6%)	3 (11.1%)	0.169
Hypertension, n (%)	134 (77.5%)	23 (85.2%)	0.363
Diabetes, n (%)	48 (27.7%)	8 (29.6%)	0.839
Smoking, n (%)	76 (43.9%)	15 (55.6%)	0.259
Hyperlipidemia, n (%)	68 (39.3%)	9 (33.3%)	0.553
Coronary artery disease, n (%)	25 (14.5%)	2 (7.4%)	0.319
Peripheral artery disease, n (%)	12 (6.9%)	2 (7.4%)	0.929
National-Institute-of-Health-Stroke-Scale-Score, median (IQR)	3 (1;5)	4 (2;9)	0.690
APB/d, median (IQR) [*n* = 194]	40 (10;220)	390 (76;1280)	< 0.001
longest SV-run, median (IQR) [*n* = 197]	0 (0;7)	6 (0;16)	0.006
Excessive-supraventricular-ectopic-activity, n (%) [*n* = 194]	30 (17.3%)	11 (40.7%)	0.003
LAD, mm, median (IQR) [*n* = 141]	39 (36;43)	46 (41;47)	0.029
LAVI, mm/square meter (m^2^), median (IQR) [*n* = 91]	33.3 (26.6;43.5)	39.0 (36.1;55.6)	0.019
LAVI/a’, median (IQR) [*n* = 75]	3.3 (2.2;4.6)	6.2 (3.5;7.4)	0.026
BNP (baseline), pg/ml, median (IQR) [*n* = 187]	28 (13;59)	58 (35;194)	< 0.001
lacunar syndrome, n (%)	36(20.8%)	3(7.7%)	0.113
territorial lesion, n (%) [*n* = 198]	69 (49.4%)	20 (74.1%)	0.001
TOAST-classification: LAA or CE or cryptogenic (vs. SAO/other), n (%)	122 (70.5%)	23 (85.2%)	0.163

Patients with and without detected pAF. Numbers are counts (percentage), median (interquartile range). Sample sizes are given in square brackets in case of missing values

Prolonged SV-runs, frequent APB, elevated BNP levels, and increased LAD showed relevant differences between those with and without AF, whereas suspected etiology expressed by the TOAST-classification and age showed no relevant differences.

**Fig. 1:**
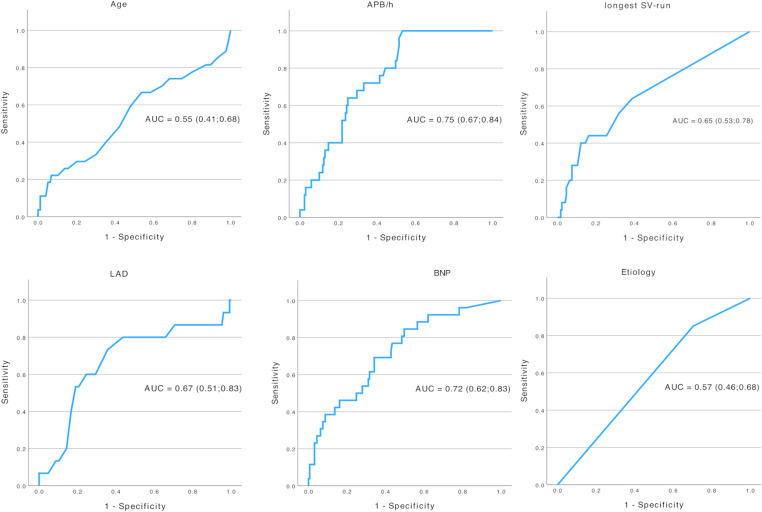
ROC-analyses of AF-predictors. ROC-curves and AUCs for age, APB, SV-runs, LAD, BNP, and suspected stroke etiology

ROC analyses resulted in an AUC of 0.55 [95%-confidence interval, CI 0.41–0.68] for age, 0.75 [0.67–0.84] for APB, 0.65 [0.53–0.78] for SV-runs, 0.67 [0.51–0.83] for LAD, 0.72 [0.62–0.83] for BNP and 0.57 [0.46–0.68] for suspected stroke etiology (see Fig. 1).

Additional ROC-analyses of more elaborate echocardiographic measurements resulted in an AUC of 0.70 [95%-CI 0.56–0.85] for LAVI (available in 91, 45.5% of all patients) and of 0.74 [0.53-0.0.95] for LAVI/a’ (available in 75, 37.5%), see Supplemental Figure S1.

**Fig. 2:**
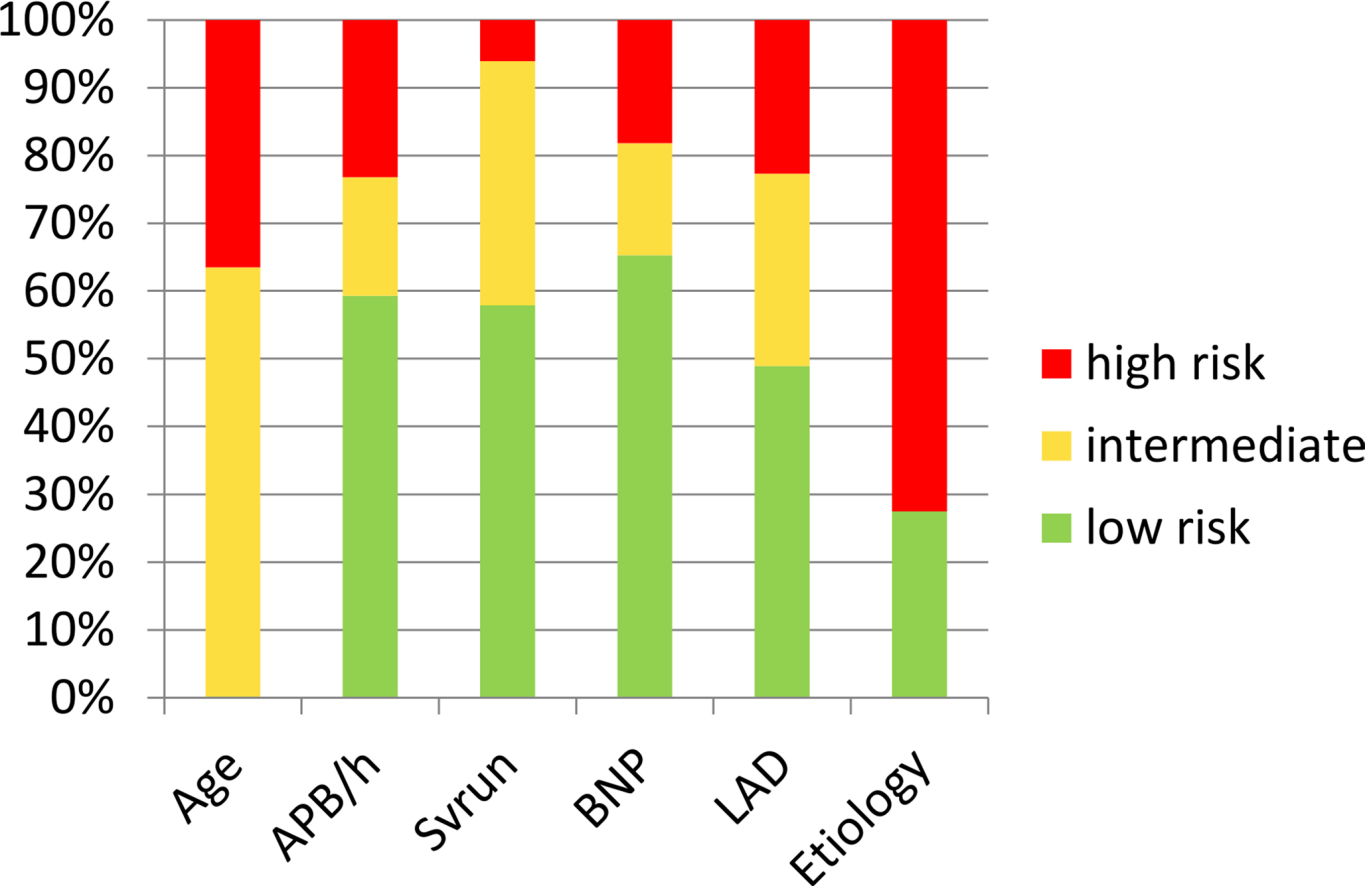
Patient numbers within risk groups. Cut-offs for predefined AF-predictors, patients per subgroup, low-risk=green, intermediate-risk=yellow, high-risk = red

**Fig. 3:**
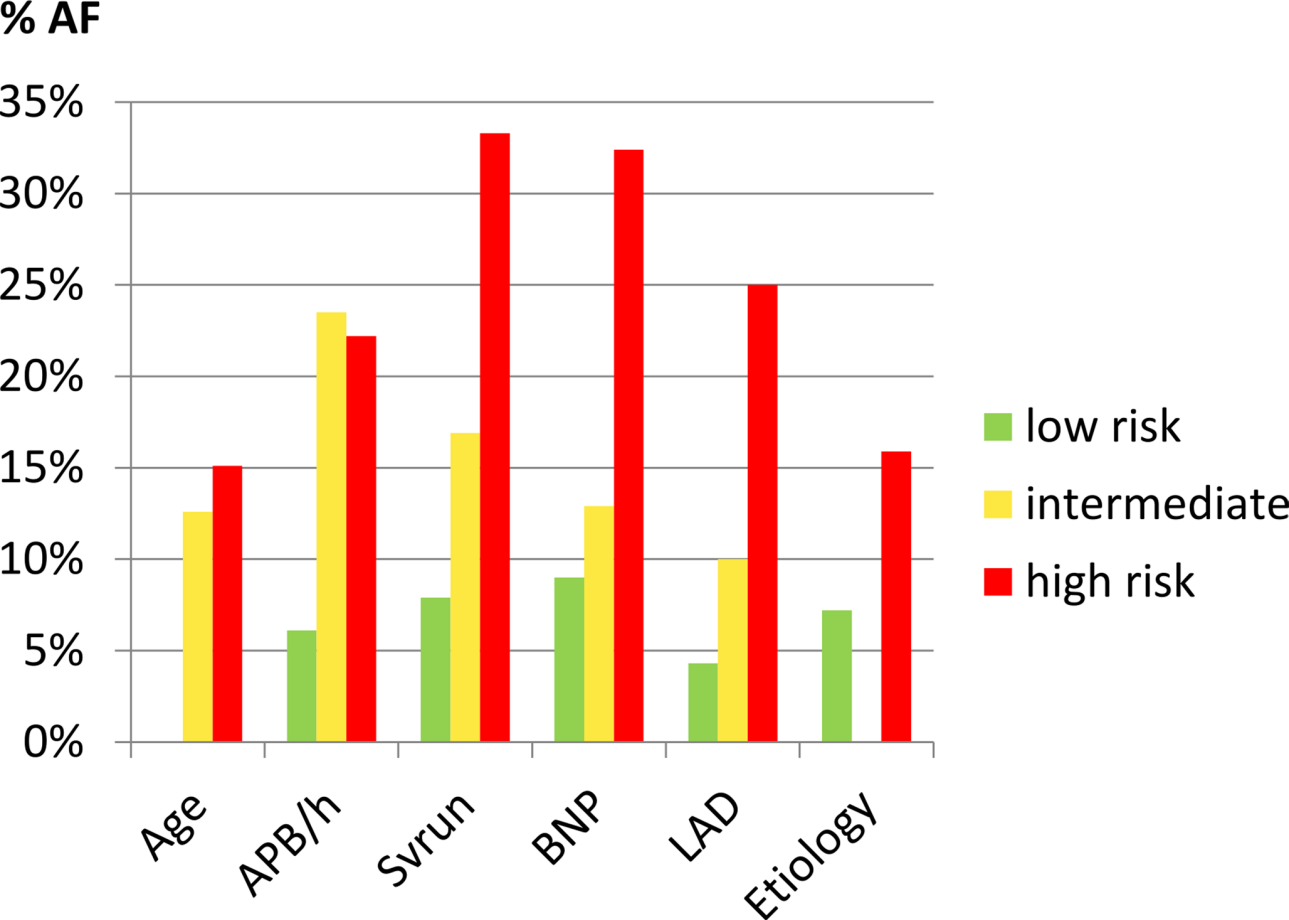
**A**F-detection rate within risk groups. AF detection-rate within each cluster, low-risk=green, intermediate-risk=yellow, high-risk = red

We applied the pre-specified cut-offs to determine a low and high risk of underlying AF. Figure 2 shows the proportion of patients and Fig. 3 shows the AF-detection rate in each cluster.

The Sensitivity, specificity, positive and negative predictive values for the pre-defined cut-off levels are shown in Table 2.

**Table 2:** Predefined AF-predictors with cut-off levels

	Cut-off	AF-prevalence *n* (%)	Sensitivity	Specificity	Positive predictive value	Negative predictive value
**Predictor variable**						
Age	< 60 years	Not available				
	≥ 60 years	16/127 (12.6)	Not available			
	≥ 75 years	11/73 (15.1)	47.2%	62.2%	11.0%	92.1%
APB	< 120/day	7/115 (6.1)				
	≥ 120/day	8/34 (23.5)	72.0%	63.9%	22.8%	93.9%
	≥ 480/day	10/45 (22.2)	40.0%	79.3%	22.2%	89.9%
SV-runs	no SV-runs	9/114 (7.9)				
	≥ 5 beats	12/71 (16.9)	64.0%	61.0%	19.3%	92.1%
	≥ 20 beats	4/12 (33.3)	16.0%	95.3%	33.3%	88.6%
LAD	< 40 mm	3/69 (4.3)				
	≥ 40 mm	4/40 (10.0)	77.3%	50.6%	11.9%	96.3%
	≥ 45 mm	8/32 (25.0)	54.5%	80.8%	19.7%	95.4%
BNP	< 50pg/ml	11/122 (9.0)				
	≥ 50pg/ml	4/31 (12.9)	59.3%	70.6%	23.1%	90.9%
	≥ 100pg/ml	11/34 (32.4)	40.7%	86.5%	45.8%	90.2%
Suspected etiology	SAO/other	4/55 (7.3)				
	LAA, CE or cryptogenic	23/145 (15.9)	83.3%	31.2%	10.8%	95.0%
**High risk factors**						
	0	1/26 (3.8)				
	≥ 1	6/69 8.7)	96.3%	14.5%	14.9%	96.2%
	≥ 2	7/60 (11.7)	74.1%	50.9%	19.0%	92.6%
	≥ 3	7/31 (22.6)	48.1%	81.5%	28.9%	91.0%
	≥ 4	5/12 (41.7)	22.2%	95.4%	42.9%	88.7%
	≥ 5	1/2 (50.0)	3.7%	99.4%	50.0%	86.9%

AF detection within each cluster of predictors and according to the number of fulfilled high-risk criteria. Sensitivity, specificity, positive and negative-predictive-value given for those fulfilling each criterion compared with those below the cut-off

SV-runs ≥ 20 beats showed the highest specificity (95.3%) and positive-predictive-value (33.3%), followed by BNP ≥ 100pg/ml (85.7% and 32.4%). The subgroup with an LAD<40 mm had the lowest AF-detection rate (4.3%). The positive-predictive-value increased with every fulfilled high-risk criterion.

The full regression model (see Supplemental Table 1 A) included 131 of all 200 patients (13 with AF) for whom data for all parameters were available. The highest odds ratios (OR) were found for those with prolonged SV-runs ≥ 20 beats (vs. 5–19 beats: OR = 6.50 [95%-CI: 0.78–53.85] and vs. no SV-runs: 12.73 [1.22-133.28]) and those with BNP>100pg/ml (vs. 50-100pg/ml: OR = 14.10 [95%-CI 1.20-166.17] and vs. <50pg/ml: 7.46 [1.52–39.42]), see Supplemental Table 1SB. After stepwise selection, BNP (50–100 vs. <50pg/ml: OR 0.70 [95%-CI: 0.16–3.13] and ≥ 100 vs. <50pg/ml: 4.45 [1.67–11.83]) and SV-runs (5–19 vs. no SV-runs: OR 2.20 [0.85–5.73] and > 20 vs. no SV-runs: 9.09 [1.79–46.05]) remained independent AF-predictors (see Supplemental Table S2).

## Discussion

The major finding of this post-hoc analysis of data from the Find-AF_RANDOMISED_-trial is that increased plasma levels of BNP and prolonged SV-runs but also left atrial enlargement and frequent APB, are useful predictors of paroxysmal AF in stroke patients. On the other hand, the suspected stroke etiology according to the TOAST classification and increased age showed no relevant predictive value within our cohort. In multivariable analyses, prolonged SV-runs and elevated BNP were independently associated with AF. Thus, AF could be linked to structural and electrical atrial alterations, which probably lay the foundation of developing AF and affect the likelihood of its onset (and therefore the AF-burden) in individually varying degrees.

ESVEA has been shown to be predictive of incident atrial fibrillation in the population-based “Kopenhagen-Holter-Study” [6], in patients with arterial hypertension [33] and various stroke populations [9, 10, 12, 32, 40, 43]. SV-runs remained an independent AF-predictor in our multivariable model. The cut-off ≥20 beats resulted in a high specificity and defined a subgroup with a high risk of underlying AF. There are data suggesting that APB and SV-runs can carry independent predictive value for underlying AF [43], however, we assume in this model APB did not remain predictive because of multicollinearity regarding atrial electric alterations.

Natriuretic peptides have been established as a valid predictor not only of heart failure, but of incident AF [14]. This has been established in various other studies examining stroke [3, 19, 23, 36]- and non-stroke patients [21, 24–26, 28, 33]. In our analysis BNP-levels ≥100pg/ml identified a subgroup with a high risk of underlying AF. Notably, the sensitivity was considerably higher compared with the cut-off SV-runs ≥20 beats. Left atrial enlargement on echocardiography is another established predictor of underlying paroxysmal AF in stroke [7, 27, 30]. Increased left atrial diameter showed predictive value in univariate analyses but did not remain independently predictive in the multivariable analysis in our trial. We have two possible explanations for this finding: (1) Although some data show independent predictive value of natriuretic peptides and echocardiographic measurements in large cohorts [21], both markers may still incorporate similar information, as natriuretic peptides are released due to myocardiocyte stress by atrial volume overload or increased pressure [14], which are important factors in the development of atrial enlargement. Thus, both markers probably showed multicollinearity regarding structural atrial alterations. (2) There was a high proportion of missing values as echocardiography was not mandatory according to the study protocol: One dimensional measurement of left atrial diameter (which is a relatively poor estimate of left atrial size) was available in 70% of patients and three-dimensional measurement (LAVI) in only 45%. Therefore, missing information on left atrial size may be another potential explanation. The limited availability of echocardiography in the diagnostic workup after stroke is a common issue, seen in other clinical trials [20] and in clinical practice [22]. More sophisticated measurements, such as the inclusion of Tissue Doppler Imaging measurements (LAVI/a’) may have increased diagnostic predictive value but were available in an even smaller proportion of patients.

There is a well-established correlation between age and AF incidence, both in the general population [45] and in stroke patients [3, 32, 37]. The limited predictive effect of age in our cohort is probably explainable by the fact that patients <60 years were excluded and the proportion of old-aged patients ≥80 years was small (15.5%), so that this finding should be interpreted with caution. In the observational forerunner trial Find-AF [29], which contained no age-limit, 7-day ECG-monitoring was initiated within 24h of presentation and patients presenting with AF were followed. Age not only correlated with the overall AF-detection rate, but also with more sustained AF-episodes that were detectable by 12-lead ECG or telemetry. In the Find-AF_RANDOMISED_-trial the study Holter-ECG was initiated after a mean 4 days. Due to this delay, some elderly patients with prolonged AF-episodes may have been excluded, if AF was detected by clinical standard procedures prior to randomization.

Overall, focusing on indicators of structural and electrical atrial alterations was a more promising approach than regarding the suspected stroke etiology based on the TOAST-classification. This finding is corroborated by other studies with continuous ECG-monitoring which could not associate AF with specific brain lesion patterns [4] and showed similar AF-detection rates in patients with LAA- and SAO-strokes [5]. In patients with LAA or SAO, the coexistence of AF is explainable by overlapping risk factors, for example hypertension and diabetes which also cause cardiac pathologies associated with AF. Whether further characterizing patients with cryptogenic strokes by applying ESUS-criteria improves AF-prediction remains to be determined. However, a secondary analysis of the NAVIGATE-ESUS trial found that unlike the overall study population, patients with left atrial enlargement (a potential surrogate of manifest AF) actually benefitted from oral anticoagulation [13]. This could further underline the importance of cardio-specific risk markers regarding the probability of underlying AF.

The ongoing Find-AF 2 trial [34, 42] is currently investigating a risk-adapted approach concordant with our findings: in the intervention arm of the trial prolonged SV-runs are used to assign patients to continuous ECG-monitoring by an implantable loop recorder, while those without receive 7-day Holter-ECGs. Patient recruitment has been completed, and results are expected in 2027.

### Limitations and strengths

We must acknowledge some limitations. The overall sample size and event rate are low, thus limiting the power for multivariable adjustment. As stated above, there are some missing values, especially echocardiographic data (available in only 70%), whose predictive effect may have been underestimated. There was also a limited proportion of MRI-scans (109 patients, 54.5%), which may have improved the validity of cerebral imaging and have affected clinicians’ judgements concerning the most likely stroke etiology. Furthermore, applying the ESUS-criteria may have improved AF-prediction within the subgroup of cryptogenic strokes. We only measured BNP-levels and cannot provide definite answers concerning NT-proBNP. Our data are restricted to patients ≥ 60 years and we cannot draw valid conclusions for younger stroke patients. Furthermore, our cohort consisted of Caucasian Europeans and results may differ in other ethnicities.

A strength of our analysis is that all data were collected prospectively within a randomized and controlled trial. We can provide lengthy and thoroughly analyzed ECG-data from a large cohort of well characterized stroke patients with an extensive followed-up.

### Summary

In conclusion, prolonged SV-runs and elevated brain natriuretic peptide were the most helpful predictors to identify stoke patients with a high risk of paroxysmal atrial fibrillation in our cohort. Future diagnostic studies should consider these parameters indicating atrial electric or structural alterations to distribute intensified heart rhythm monitoring, rather than focusing on the suspected stroke etiology.

## Supplementary Information

Supplementary Material 1

Supplementary Material 2

## Data Availability

The datasets used and/or analyzed during the current study are available from the corresponding author on reasonable request.

## Abbreviations

%: Percent
AF: Atrial fibrillation
APB: Atrial premature beats
AUC: Area-under-the-curve
BNP: Brain natriuretic peptide
CE: Cardioembolic
CI: Confidence interval
CT: Computer tomography
ECG: Electrocardiogram
ESUS: Embolic stroke of unknown source
ESVEA: Excessive supraventricular ectopic activity
i.e.: Id est, that is
IQR: Inter-quartile range
LAA: Large artery atherosclerosis
LAD: Left atrial diameter
LAVI: left atrial volume index
ml: Milliliter
mm: Millimeter
MRI: magnetic resonance imaging
n: number
NT-proBNP: N-terminal pro brain natriuretic peptide
pAF: Paroxysmal atrial fibrillation
pg: Picograms
OR: Odds ratio
ROC: Receiver-operator-characteristics
SAO: Small artery occlusion
SV-runs: Supraventricular runs
TOAST: Trial-of-ORG-10172 in Acute-Stroke-Treatment
