# Supplementary figures and images for: Evaluation of predictors indicating paroxysmal atrial fibrillation in patients with acute ischemic strokes: the Find-AFRANDOMISED trial
Source: Neurol Res Pract. 2026 Feb 23;8(1):12. doi: 10.1186/s42466-026-00471-x (PMC12931031; doi:10.1186/s42466-026-00471-x)

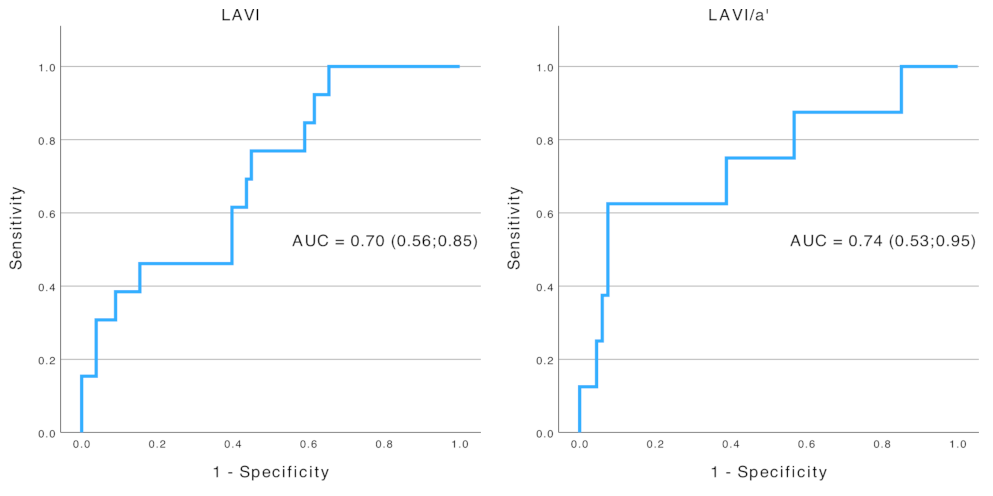

Supplement: Supplementary file 1 — Supplementary Material 1 [file 42466_2026_471_MOESM1_ESM.png]
