# Supplementary material for: Evaluation of predictors indicating paroxysmal atrial fibrillation in patients with acute ischemic strokes: the Find-AFRANDOMISED trial
Source: Neurol Res Pract. 2026 Feb 23;8(1):12. doi: 10.1186/s42466-026-00471-x (PMC12931031; doi:10.1186/s42466-026-00471-x)
Supplement: Supplementary file 2 — Supplementary Material 2 [file 42466_2026_471_MOESM2_ESM.docx]

Supplement: Multivariate models using categorical predictor variables applying cut-offs defined by the German Stroke Society

Model 1, logistic regression without selection: 131 / 200 cases in the intervention group used, AF = 13, no AF = 118.

Supplemental Table S1A:

| Type 3 Analysis of Effects | | | |
| --- | --- | --- | --- |
| Effect | DF | Wald Chi-Square | Pr > ChiSq |
| age | 1 | 0,5141 | 0,4734 |
| APB | 2 | 2,8983 | 0,2348 |
| SVrun | 2 | 4,5683 | 0,1019 |
| LAD | 2 | 2,2285 | 0,3282 |
| BNP | 2 | 7,4101 | 0,0246 |
| Etiology | 1 | 1,2341 | 0,2666 |

Supplemental Table S1B Differences of least squares means: multiple comparisons by Tukey-Kramer test.

| predictor | Pairwise comparison | | Odds Ratio Estimates | | | p-value |
| --- | --- | --- | --- | --- | --- | --- |
|  |  |  | 95% Confidence limits | | Odds ratio |  |
| age | ≥ 75 years | 60-74 years | 0.3857 | 7.7813 | 1.7324 | 0.4734 |
| APB | 120-479 APB/d | < 120 APB/d | 0.7351 | 31.2410 | 4.7921 | 0.1014 |
|  | ≥ 480 APB/d | 120-479 APB/d | 0.2492 | 7.7426 | 1.3890 | 0.7078 |
|  | ≥ 480 APB/d | < 120 APB/d | 0.5470 | 21.7628 | 3.4507 | 0.1875 |
| SV-runs | SV-runs 5-19 | no SV-runs | 0.3845 | 9.9906 | 1.9598 | 0.4181 |
|  | SV-runs ≥ 20 | SV-runs 5-19 | 0.7835 | 53.8503 | 6.4977 | 0.0829 |
|  | SV-runs ≥ 20 | no SV-runs | 1.2159 | 133.28 | 12.7304 | 0.0337 |
| LAD | LAD 40-45 mm | LAD < 40 mm | 0.4316 | 18.9681 | 2.8613 | 0.2760 |
|  | LAD > 45 mm | LAD 40-45 mm | 0.2309 | 7.3964 | 1.3067 | 0.7623 |
|  | LAD > 45 mm | LAD < 40 mm | 0.6318 | 22.1239 | 3.7383 | 0.1460 |
| BNP | BNP 50-100 pg/ml | BNP < 50 pg/ml | 0.1834 | 18.0766 | 1.8208 | 0.6088 |
|  | BNP > 100 pg/ml | BNP 50-100 pg/ml | 1.1972 | 166.1682 | 14.1044 | 0.0355 |
|  | BNP > 100 pg/ml | BNP < 50 pg/ml | 1.5228 | 39.4166 | 7.459 | 0.0136 |
| Etiology | Large artery disease, cryptogenic, cardioembolic | Small artery disease or other | 0.4045 | 26.4098 | 3.2684 | 0.2666 |

Model 2: Logistic regression with selection in the intervention arm. 186 / 200 cases used, AF = 25, no AF = 161.

Supplemental Table S2

Selection of the two independently predictive markers BNP and SV-run.

| predictor | Pairwise comparison | | Odds Ratio Estimates | | | p-value |
| --- | --- | --- | --- | --- | --- | --- |
|  |  |  | 95% Confidence limits | | Odds ratio |  |
| SV-runs | SV-runs 5-19 | no SV-runs | 0.845 | 5.725 | 2.199 | 0.106 |
|  | SV-runs ≥ 20 | no SV-runs | 1.793 | 46.052 | 9.087 | 0.008 |
| BNP | 50-100 pg/ml | < 50 pg/ml | 0.157 | 3.133 | 0.702 | 0.643 |
|  | > 100 pg/ml | < 50 pg/ml | 1.673 | 11.828 | 4.449 | 0.003 |
